# Workplace restructurings in intervention studies – a challenge for design, analysis and interpretation

**DOI:** 10.1186/1471-2288-8-39

**Published:** 2008-06-13

**Authors:** Ole Olsen, Karen Albertsen, Martin Lindhardt Nielsen, Kjeld Børge Poulsen, Sisse Malene Frydendal Gron, Hans Lennart Brunnberg

**Affiliations:** 1 National Research Centre for the Working Environment, Lersø Parkallé 105, DK-2100 Copenhagen, Denmark; 2 Clinic of Occupational Medicine, Hillerød Hospital, Helsevej 2, DK-3400 Hillerød, Denmark; 3 Child Health Promotion, Odsherred Municipality, Vesterlyngvej 8, DK-4500 Nykøbing Sjælland, Denmark; 4 Center of Maritime Health and Safety, University of Southern Denmark, Niels Bohrs Vej 9, DK-6700 Esbjerg, Denmark; 5 Stockholm Center for Public Health, Stockholm County Council, PO box 17533, SE-118 91 Stockholm, Sweden; 6 Department of Public Health Sciences, Karolinska Institute, SE-171 76 Stockholm, Sweden

## Abstract

**Background:**

Interventions in occupational health often target worksites rather than individuals. The objective of this paper is to describe the (lack of) stability in units of analysis in occupational health and safety intervention projects directed toward worksites.

**Methods:**

A case study approach is used to describe naturally occurring organizational changes in four, large, Nordic intervention projects that ran 3–5 years, covered 3–52 worksites, cost 0.25 mill–2.2 mill €, and involved 3–7 researchers.

**Results:**

In all four cases, high rates of closing, merging, moving, downsizing or restructuring was observed, and in all four cases at least one company/worksite experienced two or more re-organizations during the project period. If individual worksites remained, ownership or (for publicly owned) administrative or legal base often shifted. Forthcoming closure led employees and managers to seek employment at other worksites participating in the studies. Key employees involved in the intervention process often changed.

**Conclusion:**

Major changes were the rule rather than the exception. Frequent fundamental changes at worksites need to be taken into account when planning intervention studies and raises serious questions concerning design, analyses and interpretation of results. The frequent changes may also have deleterious implications for the potential effectiveness of many real life interventions directed toward worksites. We urge researchers and editors to prioritize this subject in order to improve the quality of future intervention research and preventive action.

## Background

There is an urgent need for studies that look at the feasibility and effectiveness of interventions at the workplace [1]. Psychosocial and organizational circumstances have been identified as risk factors for lack of wellbeing, poor health, sickness absence and early retirement. Contrary to traditional risk factors these risk factors characterize the workplace rather than the individual employees. Interventions must thus be targeted toward the workplace rather than the individual. Similarly, intervention studies must be designed so that the workplaces are the units of intervention (and control) and also the units of statistical analysis. This will create new challenges for design and analysis of intervention effectiveness studies, irrespective of the specific nature of the intervention. However, if some interventions can be demonstrated to be effective, implementation of them may carry a large public health impact as well as a large economic impact. Thus several large-scale intervention studies have been implemented. In this paper we first briefly describe the recent history of occupational intervention research and then report on one challenging aspect of design not previously systematically reported: the stability of worksites during the years-long period of recruitment of workplaces, intervention and follow-up.

### Occupational intervention research

The importance of intervention effectiveness research in occupational health and safety was increasingly recognized in the mid 1990's. In 1995 NIOSH in the United States prioritized it as one of their topics at The National Occupational Research Agenda [2,3]. During 1997–99 the Nordic Council of Ministers sponsored a series of meetings of occupational intervention researchers to develop intervention research in the Nordic countries [4]. At about the same time a similar need for development of intervention research methodology was recognized in relation to organization and practice of care [5] and public health [6-8].

It was generally agreed that although "randomized, controlled trials are the accepted standard for determining cause and effect between interventions and outcomes ... in the context of occupational safety and health, such studies are sometimes not feasible because of practical, ethical, legal or other constraints" [9]. Other options were discussed including controlled before-and-after trials and interrupted time series [5,10,11]. To a large extent focus was on whether allocation was random or not. Stability of the unit of allocation was not discussed.

Unit of analysis is a well known statistical concern when individuals are recruited and followed in clusters and the problem may be mentioned in the section on statistical analysis e.g. "If an intervention is targeted at changing a workplace, rather than individuals, then each workplace might count as just one unit" [11]. However, whereas individuals are well-defined entities throughout intervention and follow-up periods, "workplaces" may turn out to be more ambiguous and less stable entities.

Empirical experience with planning and analysis of cluster randomized trials in occupational health seems limited. In the introduction to the CONSORT guidelines [12] five critical reviews are mentioned covering in total more than 200 reports of cluster randomized trials. The clusters typically refer to schools, families, villages, and patients of individual physicians but only in three identifiable cases to worksites. In a review of 45 health promotion trials at worksites only 11 studies reported worksite participation rates [13]. Thus even when workers at specific worksites have been followed, the stability of the worksites has rarely been reported. Empirically documented evidence of the stability of worksites over time seems limited.

Occupational organizational intervention studies face many other difficulties with respect to design, implementation and evaluation. Following a decade of experiences some of these difficulties (e.g. lack of understanding of the implementation process, treatment diffusion, etc) were described in text book chapters and review papers [14-17]. These other problems are not the focus of our paper. On the other hand, the potential problem with lack of stability of worksites over time was not specifically mentioned in the text books and review papers. In the most recent paper, it was stated that "The problem is that many ... factors are often not reported at all ... Or they are reported in a more or less anecdotal way", and "careful documentation of as much information as possible in a systematic fashion... could take the interpretations by authors out of the realm of anecdotes and support them with data" [17]. The aim of our paper is to report such data on the stability of units of intervention in large occupational health and safety intervention projects in a systematic way.

## Methods

Several intervention studies were presented at the first international course on intervention research, held by the Nordic Institute for Advanced Training in Occupational Health, September 2004. The first author of the present paper had been involved fairly late as statistician in an intervention project that was not presented; at that time he had been puzzled by the many stories told by the involved interventionists about the events at the individual workplaces in the study and his own difficulties in keeping track of intended and unintended events in a traditional cause and effect analysis. While listening to the presentations of the many other intervention projects at the course, he had a feeling of déjà vu and asked the lead interventionists to join and prepare a paper presenting the "dissolving-unit-of analysis" aspect of their studies. The four examples presented in this paper are thus a convenience sample of four Nordic studies set up during the late 1990's, brought together for illustrative purposes. Except for the retrospective nature of our study, our approach is very similar to Yin's recommendations for analysis and reporting of descriptive, multiple case studies [18].

Duration, size and cost of the projects are summarized in Table 1. Studies are presented in order of increasing complexity. The third, fourth and fifth columns describe the size of the projects: number of companies, number of worksites and number of participants. Thus, the individual level is opposed to two logical levels of workplaces: worksites and companies. So far we have used the terms workplace and worksite interchangeably. From now on we will use the term worksite to identify the geographically located worksites and the term workplace as an umbrella term for both worksites and the larger administrative units labeled companies in Table 1. Most examples in this paper refer to worksites. Additional details for each study regarding aim, start-up, and design are given in the ensuing four subsections; the multi-facetted nature of the interventions is briefly described. References to our studies include non-peer-reviewed reports when these give the most detailed description of the project.

**Table 1:** Duration, size and costs of four occupational intervention projects.

	Project period	Companies/organisations/municipalities involved	Worksites	Participants	Research Costs	Researchers employed^§^
Stockholm bus drivers	1999–2002	2 companies	3 garages	640 employees	0.25 mill. €	2 (+1)
Copenhagen bus drivers	1998–2003	6 companies at start	20 garages at start	3505 at baseline	2.2 mill. €*	1^|| ^(+6)
Women at work	2000–2004	21 companies/municipalities	31 at baseline	2183 at baseline	2.1 mill. €^†^	3^¶ ^(+4)
Intervention Project on Absence and Well-being	1996–2001	2 municipal organisations 1 private company	52 at baseline	2730 employees 1919 respondents	0.5 mill. €^‡^	1 (+2)

* The direct research costs.

^† ^Half the funding was for research and evaluation, the other half for the interventions.

^‡ ^Intervention activities were financed by the companies. The municipal workplaces used approx. 0.3 million Euro for external process consultants. All workplaces used money for activities and parts of the staffs working time for meetings, working groups etc.

^§ ^Numbers in parentheses indicate senior researchers, researchers and research assistants working less than half time on the project or PhD students funded by the project.

^|| ^The project leader was a senior researcher, employed full time throughout the project, except for one year on leave during the 3^rd ^and 4^th ^year of the project.

¶Two research assistants were working on the project throughout the period but the project manager changed in the middle of the project period.

Unintended changes are described for each project in the results section. For the two least complex cases the descriptions are based on concurrent information from managers and other participants about events that might influence the project, information from meetings, reading of company journals, union journals, news papers and general record keeping by the involved researchers; in addition, in the Copenhagen busdrivers project a common electronic log of all sorts of important events that might influence the project (in total 60 pages of text) were kept. The figures that illustrate the events and changes are slightly modified and translated power point slides from presentations of the studies; the texts accompanying the figures are elaborated versions of the accompanying talks. For the two more complex cases no single power point slide could be transformed into a useful figure. The tables were created based on information extracted from reports in Danish. The first author (OO) collaborated with MLN in extracting and tabulating data from a report in Danish (100 pages) in turn based on interviews and a plethora of individual reports related to the sub-projects; KA extracted data from 13 reports relating to the individual intervention projects (each report approximately 15 pages, in total appr. 200 pages) and made a first complex draft table that was modified and simplified in collaboration with OO. Thus the level of detailing is not entirely similar for our four cases. However, stability of unit of intervention and change of key personnel is covered for all projects.

### Stockholm bus drivers [19]

The aim of the study was to develop and evaluate a strategy for improving the psychosocial work environment of bus drivers, for improving the work environment of the three involved worksites, and finally for diffusing the strategy to other worksites in the same industry. The project was initiated by Center of Public Health in order to improve bus drivers' poor work environment in the Stockholm area. As the study was developmental and did not aim at measuring cause and effect, the individual busdrivers were not interviewed and followed. The interventionists worked with managers, drivers and other relevant persons in working groups each focusing at one specific problem and an umbrella group deciding which problems to prioritize (eg. more realistic time tables, improved communication, etc).

### Copenhagen bus drivers [20-22]

The aim was to involve drivers and owners in multi-facetted, mutually binding health promotion projects. The idea for the project was born following several national studies showing that bus drivers were among the jobs with the highest cardiovascular mortality and other disease burdens. The project was kick-started by a conference involving the ministers of labor and of traffic, the unions, the bus owner association and the National Institute of Occupational Health. The project involved all bus companies in the Greater Copenhagen Area. Baseline and two follow-up surveys were conducted with two years in-between. The intended design of the study was a before-and-after study with the garages as the individual units of intervention.

### Women at work [23-25]

The over-arching aim of the project was to prevent absenteeism and exclusion from the labor market, particularly among women. The two specific goals of the program were: i) to identify working conditions improving or reducing the well-being and work capacity of women and ii) to initiate and evaluate organizational-level interventions to improve the psychosocial and ergonomic environment and promote employee health. The program was initiated and funded by the Danish Ministry of Employment. The specific content and target of the intervention projects varied from organization to organization, but all had the same overall objectives. The interventions were initiated and implemented by worksites in collaboration with consultants. The program was organized as a multiple case intervention study, with matched comparison groups. The evaluation of the program and research part was conducted by researchers. Baseline and follow-up surveys were conducted with a 20-month interval in each intervention project.

### Intervention Project on Absence and Well-being (IPAW) [26-28]

The aim of this project was to investigate if interventions to improve the psychosocial work environment could improve the employees' well-being and health, and reduce absence from work. The project was initiated in cooperation between researchers at NIOH, Denmark and occupational physicians at the occupational health services affiliated with the workplaces. The design intended to compare the development at similar workplaces, e.g. nursing homes, with and without interventions to actively improve psychosocial workplace factors like decision authority, skill discretion, psychological demands, support from leader and colleagues, meaningfulness of work, and predictability. Interventions were intended to take place at 22 worksites matched to 30 control worksites in municipal care (13 intervention worksites + 9 control worksites), municipal technical services (4+13) and a large pharmaceutical company (5+8). Interventions were supported by process-consultants and were performed during 1996–98. Data collection for follow-up continued until 2001.

### Ethics

The Danish studies did not need ethics committee approval. An application relating to the first Danish study (IPAW) was sent to the ethics committee but was returned with the explanation that it did not need approval as it was not clinical or biomedical research; similarly for the second study (Copenhagen Busdrivers); for the third (Women at Work) no application was sent because no biological samples were taken and no sensitive questions were asked in questionnaires or interviews. The Swedish study was approved as a matter of precaution by Lokal forskningsetikkommitté Nord, Karolinska sjukhuset with accession number 97–162.

## Results

### Stockholm bus drivers [19]

**Figure 1:**
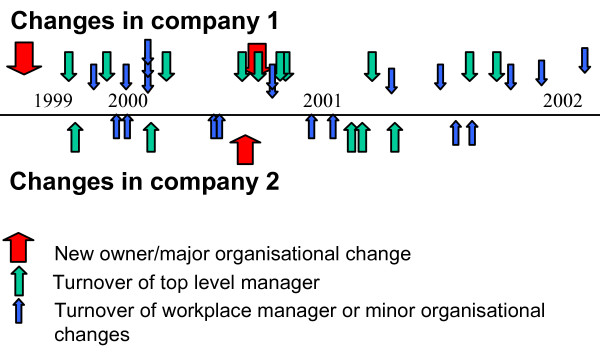
Naturally occurring organisational or managerial changes in the Stockholm bus project during project period.

It turned out that the aims of improving the work environment and diffusing the strategy was difficult to achieve. The main reason for this was an ever-changing organization at all levels of the bus companies (see Figure 1). In order to attain results at the individual worksites and then to diffuse them throughout the bus company, support was needed from managers at all levels. There was considerable turnover at the managerial level, and substitutes were often brought in. The new managers were unable to back up the suggested improvements due to their lack of experience with the project. Information about the project to these new managers was of little help, since they often did not have sufficient time to gain firsthand experience of the project.

Furthermore, two changes of owners and a further major reorganization resulted in a temporary loss of interest in the project from the top and the middle management levels. The project continued during the period of these changes, but only on a part-time basis.

Finally, some minor changes at the worksites led to problems. One example was the practice of using selected bus drivers to spread information to their colleagues. The researchers used these informants to inform the staff about developments in the project as well. This practice was discontinued after one year due to a new top level manager, who abolished the informants to reduce costs, with one result being much less information about the project reaching all the drivers.

### Copenhagen bus drivers [20-22]

**Figure 2:**
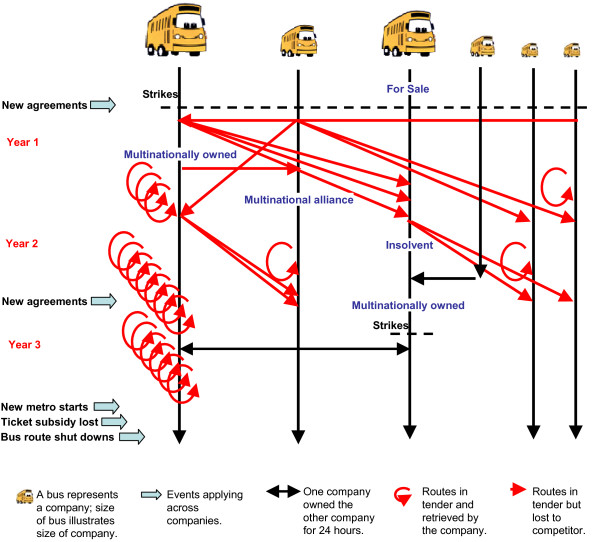
Naturally occurring major changes in bus companies in Greater Copenhagen Area during three-year period.

Just prior to the study period the national and the regional public bus operators that ran most lines in the study area were made ready for privatization. During the first three years of the project period, the previously public companies were sold, mainly to large international transport corporations, but parts were also traded back and forth between companies. Every year 12% of the lines were in tender. This had great impact on the organizational structure and company culture. The larger companies changed ownership one or more times during the planning and intervention phase, whereas individual garages were closed, merged, moved or sold, with or without all or some of the drivers previously operating from the garage, while individual lines serviced by a garage or company following tendering might move to be serviced from another garage or company during planning, intervention and follow-up period (see Figure 2 for additional events). At the garage level dozens of major or minor changes took place, e.g. change of local management, shop or safety stewards or local strikes.

### Women at work [23-25]

At project start, 17 intervention projects were granted. Four of these projects were stopped after the baseline measurement but before the intervention started, one because of economical crises in the branch, two because the initiating project leader changed job, and nobody could take over, and one stopped for other reasons. Due to this dropout of projects, 2183 employees participated in the baseline measurement but only 827 in the follow-up. Two projects were organized as courses with participants coming from different organizations, and accordingly with different organizational contexts. The remaining 11 projects took place in 21 different organizations or municipalities and covered 31 different worksites or departments.

A lot of organizational changes took place in the intervention period (see Table 2 for an overview of changes at the intervention worksites. Changes at the comparison worksites are not covered). It is beyond the scope of the present paper to describe the changes in detail, but for one of the condensed entries in the table (industrial canteens) a very detailed account has been published [24], from which we quote: "At workplace A, the process evaluation highlighted several factors that affected the processes. First, the workplace had recently been through several organizational changes, e.g., fusions with other canteens, and employees reported a high level of "change fatigue." ... the implementation of the intervention was affected by the fact that the overall administration introduced a non-smoking policy at the whole workplace at the time the interventions were implemented. That caused a negative atmosphere and disagreements among the employees during the intervention phase. ... At workplace B the implementation of the interventions started at the same time as a new manager was employed. This seemed to be crucial for the implementation processes." Similar changes and co-interventions at the comparison worksites are also described in the reference.

**Table 2:** Unplanned events at intervention worksites in the Women at work project.

**Types (and numbers) of worksites**	**Changes at worksites**	**Change of key personnel**	**Other**
Municipal elderly care (1)	Several mergers	Project leader, Chief/manager	Municipal restructuring
Municipal care (2)	Closed, Downsized	Project leader	External consultant
Hospital health care (9 units)	Downsized	Chief/manager	.
Municipal health care (1)	Restructured, Merged	.	.
Library (1)	Downsized	.	.
Industrial canteens (2)	Restructured	Project leader, Chief/manager	Smoking policy
Cleaning company A (2)	.	.	Quality development process
Cleaning company B (2)	Restructured	Project leader, Chief/manager	.
Hospital cleaning (4)	Closed, Downsized	Chief/manager	.
Retail trade and industry (3)	Restructured	.	Competence development
Food industry (2)	Downsized	Project leader	Work environment screening

Many of the changes seriously affected the implementation of the interventions. The turnover and change of project leaders in some cases had the effect that responsibility and engagement vanished. The negative emotions associated with downsizings or cutbacks negatively affected the intervention climate among the participants and their interest and engagement in the projects. Organizational changes and mergers of units did in some projects interrupt substantially the intervention process. Participants who started in the intervention group, ended up in the control group due to organizational changes, and intervention group units were merged with non-intervention groups in the process.

### Intervention Project on Absence and Well-being (IPAW) [26-28]

Unanticipated changes occurred at several levels. At the top level both the municipal and the private organizations changed their structure. The pharmaceutical company divided into two separate companies, reorganized some departments, and reduced the staff at some worksites for the first time in living memory. In the municipal technical services, the organization was prepared for outsourcing and tenders, and many former units were split into buying/ordering and selling/executing parts. This caused restructuring of departments, new relations with management, and widespread fear of redundancy if private companies were to take over. Four administrative offices (1 intervention, 3 control) were so heavily restructured that it was decided to stop follow-up, as the same functions and employees could no longer be clearly identified in the new organization. In municipal care, the institutions for mentally handicapped were transferred from the health administration to the social and welfare administration, and the district structure and management were changed. A long awaited plan for rebuilding the nursing homes were finally financed during the intervention phase, and caused 2 participating nursing homes to close down during intervention and 2 during follow-up (3 intervention homes, 1 control home).

At worksite and working group levels, a wide range of changes occurred, including change of supervisor, reduced staff, altered tasks, merging, reorganization etc. Some of this was due to top-level actions, some due to local decisions. Besides the discontinuation of follow-up due to restructuring and closing, 3 of 22 intervention worksites gave up the intervention assignment due to different difficulties, but were still participating in follow-up (1 production factory in the pharmaceutical company, 1 nursing home, 1 institution).

More detailed examples from the 17 worksites in the municipal technical services are described in Table 3.

**Table 3:** Changes in 17 municipal technical service worksites during intervention project

• Five road maintenance sites were involved. During spring 1999 two of the five sites were closed down and the employees transferred to the three surviving sites, partly across intervention/control status. Autumn 1999 the remaining sites were merged with the road construction department.
• One park maintenance site was included. In 1998 the nine existing districts were merged into five. In 2000 these were merged into one unit, and redistributed into 8 "foreman areas" approximately equal to the original nine districts. In 2002 park maintenance was merged with street cleaning into "town service", sub-sectioned into five districts each with independent economy and administration.
• Two workshops (auto repair and metal works) remained undivided and unmerged but lost activities and employment due to the new intra-municipal accounting principles and preparations for tendering of activities.
• Two cemeteries and three pumping stations remained relatively stable, and were neither prepared for privatisation nor outsourced.
• Four administrative offices involved in planning and accounting of the above work had some staff moved to a central "buying" office and others distributed out to the local "selling" units as part of preparation for outsourcing. All offices were so heavily reorganised that at the end of 1998 it was formally decided to leave out the offices from follow-up in the study.

## Discussion

### Findings

We have followed more than one hundred worksites over several years and described the instability of the units of intervention. Our four intervention projects all suffered from a fairly high rate of close down, merging, physical moving or organizational restructuring of the worksites over a short span of years. If the individual worksites remained, the ownership of the private company or the administrative or legal base of the publicly owned worksite might shift. We also saw that even while worksites still formally existed, expectation or announcement of their forthcoming closure might lead – or were even intended to lead [26] – to employees seeking employment at other similar worksites. And even if ownership, address and organization were all stable (which rarely happened), the persons that we collaborated with or the persons responsible for time and resources being set aside for the interventions internally at the worksites might change one or more times.

The instabilities in the units under study give rise to different kinds of problems in the research process; the worksite under study may change so fundamentally that it is not meaningful to compare before and after measures at all; also changes of project manager or other important persons may be as detrimental to the "unit of analysis" as changes in the worksite itself and will often lead to reductions in intensity [17].

The amount and diversity of ongoing restructuring of worksites was not anticipated in the design of our intervention studies. Nor had we encountered any clear warnings in the theoretical literature on intervention studies. We therefore felt a need for this empirical report of our experiences of the incidence and diversity of such instabilities.

### Strengths and weaknesses

In line with the advice in Yin's book on case study research [18] our data analysis consisted of examination, categorization and tabulation of the evidence as well as use of the dominant analytical technique, pattern-matching. We have also avoided doing statistical analysis. It may be considered a weakness that our pattern was not "defined prior to data collection". On the other hand, the categorization and tabulation presented in this report was loosely and independently pre-specified and requested by the first author, delivered by the lead investigators of the individual projects, and to some extent modified in collaboration. In this way we are in line with the advice "not to postulate very subtle patterns", but rather to specify patterns that "are likely to lead to gross matches or mismatches and in which even an "eyeballing" technique is sufficiently convincing to draw conclusion". Nevertheless, "this lack of precision can allow for some interpretative discretion on the part of the investigator". We thus advise the reader to continue reading with a critical mind.

### External validity

Our four intervention studies are not representative; they constitute a convenience sample. We will thus not claim that they represent average worksites in any way. On the other hand, as indicated in Table 1, the studies are fairly large. All studies were represented by researchers at the First international NIVA-course on Intervention Research, September 2004, Copenhagen, Denmark, and three of the studies were presented.

It may be hypothesized that the prevalence of changes in our sample was particularly high because sectors with known occupational problems had been selected and because these problems were partly related to high rates of changes. This might apply to many (previously) public workplaces being prepared for or recently privatized and to parts of the private sector, undergoing e.g. lean management (IPAW). On the other hand changes seem to be planned at an ongoing high rate. While we were preparing this paper it was politically decided in Denmark (and to almost the same extent in Sweden) that the entire public health care sector, the police, the taxation authorities, the universities and most other public research, the municipalities and the counties were going to be completely restructured and many employees moved around. The private sector is similarly undergoing outsourcing, migration, globalization and Enronization.

We have not been able to identify other empirical studies with a similar focus on stability of worksites in intervention studies. This is supported by the recent statement that also broader evaluations of such studies are scarce in the literature [29]. A few studies, however, indicate similar findings. A Norwegian process evaluation had a broader scope than ours and focused on obstacles to implementation in general. Based on 130 interviews at the worksites and with the use of grounded theory, the researchers were led to five categories of obstacles, the fifth of which being organizational changes. Their fifth category (similar to our focus) emerged despite their different focus and despite their much shorter follow-up period (1 year). The most devastating reorganization actually occurred "a couple of weeks after the project started up" and despite the fact that the organization "had originally been selected because it was considered to be a fairly stable enterprise". The authors state "the situation was not as predictable as we had expected before the project began" [29]. The author of another Norwegian paper discussing partly the same intervention projects (overlap: postal service) state that "In the electric energy sector the results of organizational restructuring were a larger threat ... than in the Postal Service" [30]. A survey of British managers showed that around 60% had experienced some form of reorganization in the last year.[31] Thus, although the incidence and characters of organizational changes haven't been studied systematically, other studies seem to confirm a high incidence.

Stimulated by reviewers, we further considered whether our convenience sample had some common denominator that made the workplaces special and thus the external validity low. One way to assess this could be to compare with a sample of the almost 1000 occupational health intervention studies collected by The Cochrane Occupational Health Field [32]. However, we consider this task beyond the scope of our paper. As an exercise – because two of our four case studies included health care workers – we checked the original studies included in one selected Cochrane review on stress prevention in health care workers [33]. The review included three studies. One was extremely small and comprised only one intervention and one control group; we did not investigate this any further. We ordered the original publication for the two other studies [34,35]. They did not report changes systematically. However, Proctor et al indirectly indicated drop out or major changes in about a third of the workplaces, and stated in the discussion that: "Anecdotally, there were organisational and managerial changes which appeared to be causing a certain degree of distress amongst staff in a number of the homes involved in the study which may account for the observed increase in GHQ scores among staff in both the control and intervention groups" [34]. The extremely well designed second study [35] reported a very low drop-out rate (1 of 16 homes selected for randomization). However, workplaces seem to have been selected based on expectations of stability, and could not be considered representative. The authors state in their discussion: "The interviewees also mentioned that organizational problems may have hindered the implementation of the intervention"[35] with reference to a qualitative study published in Dutch. The findings of these two studies and the anecdotal (!) observation do not contradict our findings; they also demonstrate that the information is not presented in prominent places or easily accessible publications. Thus it supports our attempt to move such observations more systematically out of the realm of anecdotes and non-English publications.

It might, for comfort, be hypothesized that we are in a period of particularly much change and that things will later settle. However, the Roman prefect Gaius Petronius AD66, has been quoted for the following: "We trained hard, but it seemed that every time we were beginning to form up into teams we would be reorganized. I was to learn later in life that we were to meet any new situation by reorganization. And a wonderful method it can be for creating the illusion of progress while producing confusion, inefficiency and demoralization". So, even though the quote has been characterized as a spoof, it may date as far back as World War I [36] thus covering the entire period of modern society.

### Challenges

The above mentioned challenges may lead to the assumption that it is more difficult to set up studies of high methodological quality in occupational intervention research than in clinical medicine. However, a recent systematic review showed that less than a third of the studied 114 randomized clinical trials achieved their planned sample size and that the recruitment period was extended for around half of the trials, usually supported by a supplementary grant, leading to less reliable results [37]. In a qualitative study related to the systematic review the authors state that "Securing and managing finances ... is a highly complex activity", "the ... system of dividing funds ... brought the trial teams into complicated negotiations with multiple funders", "The fact that all funders had the potential to influence and shape the trials ... was an important issue as the perspectives of applicants and funders could diverge", and "From development to completion ..., the trialists had to be resourceful and flexible, adapting to changing internal and external circumstances" [38].

Thus intervention research seems challenging in general, whether in clinical medicine or in occupational health. Most challenges are common: constantly changing circumstances, a need for resourceful and flexible interventionists, complicated negotiations with multiple stakeholders (whether funders or worksite managers), not reaching the planned sample size (whether because of over-optimism relating to recruitment or stability of worksites), the need for additional funding and finally less reliable research results.

The trial reviewers had hoped to identify factors associated with successful recruitment to enhance the chances of success. However, their analyses "yielded few insights" [37]. In the accompanying qualitative study they call for further similar research, aimed at understanding and facilitating the conduct of clinical research [38].

In occupational intervention studies randomisation would not in itself solve the "dissolving unit of analysis" problem but an increase of the sample size with a factor 3–5 additional to the sample size suggested by a usual power calculation would give a sufficient number of stable workplaces to make conclusions about the potential effectiveness of the intervention at stable workplaces. However, for less stable workplaces additional challenges have to be solved before valid intervention studies can be effectively designed. Time and money might be saved if stakeholders in quality improvement in working life could sit together and develop research designs that could give more generalizable and reliable results" [30].

## Conclusion

Fundamental changes in the organization of worksites are more common than has been presumed. It seems as if the expectations were too high during the 1990's with regard to how often well designed longitudinal studies of organizational change might be feasible. Realistic expectations are crucial if the effects of interventions to improve productivity and working life are to be assessed in a cost effective and sufficiently valid manner.

### Implications for occupational intervention studies

Researchers should record and report stability of workplaces (ownership, mergers, moves, etc), change of key personnel (eg local middle manager, head of department, shop steward), and turn over rate of staff; this applies especially for studies running for more than a year. Editors of occupational health journals should call for publication of such information from recently finished studies as experiences from the last decade are not yet sufficiently well described. Practitioners should be aware that lack of reporting of changes, may give false credibility to useless or even harmful interventions.

### Implications for methodological research

It might be worthwhile doing a systematic review of published occupational intervention studies, noting whether naturally occuring changes have been reported, either anecdotically or in a more systematic way, and, if so, to extract the types and numbers of changes. Funders and research institutions should make room for interventionists to publish, meet and reflect on such findings regarding the real-life context in which the interventions are deemed to take place. New designs that fit real-life contexts better might be developed and tried out in practice [39] ensuring publication of more reliable and useful evidence.

## Competing interests

Five of the authors are or have been employed by NRCWE that may potentially gain or lose financially from the results of our study.

## Authors' contributions

OO suggested the paper, outlined the structure and drafted the introduction and discussion. KA, MLN, KBP, SMFG and HLB provided details about the four studies. All authors contributed to the final version and read and approved it.

## Pre-publication history

The pre-publication history for this paper can be accessed here:


